# First chloroplast genomics study of *Phoenix dactylifera* (var. Naghal and Khanezi): A comparative analysis

**DOI:** 10.1371/journal.pone.0200104

**Published:** 2018-07-31

**Authors:** Abdul Latif Khan, Sajjad Asaf, In-Jung Lee, Ahmed Al-Harrasi, Ahmed Al-Rawahi

**Affiliations:** 1 Natural and Medical Sciences Research Center, University of Nizwa, Nizwa, Oman; 2 School of Applied Biosciences, Kyungpook National University, Daegu, Republic of Korea; Chinese Academy of Medical Sciences and Peking Union Medical College, CHINA

## Abstract

Date palm (*Phoenix dactylifera* L.) is one of the oldest fruit crops in the arid regions of the Middle East. However, little information is available regarding its plastid genomes. In this study, we sequenced the chloroplast (cp) genomes of two economically important but genomically unexplored date palm cultivars of *Phoenix dactylifera* (var. Naghal and Khanezi). The data assembly and genome annotation revealed a typical quadripartite structure similar to *Arecaceae*, and the genome sizes of Naghal and Khanezi were 158,210 bp and 158,211 bp, respectively. Structurally, both cp genomes were comprised of four regions: a pair of inverted repeats (27,273 bp for Khanezi and for Naghal 27,272 bp), a large single-copy region (86,090 bp and 86,092 bp) and a small single-copy region (17,575 bp and 17,574 bp). Both genomes had 138 representative genes, whereas 227 and 229 randomly distributed microsatellites were also observed in Khanezi and Naghal, respectively. Phylogenetic analysis based on the whole cp genomes and 68 shared genes showed identical phylogenetic trees of Khanezi and Naghal forming clades with Khalas and Aseel cultivars, respectively. The current study showed detailed comparative cp genome analysis, which could be essential for broader population genetics and molecular studies of these four date palm cultivars.

## Introduction

Date palm, *Phoenix dacylifera* L., belongs to *Arecaeae* and is an ecologically, culturally and economically important fruit crops in North Africa, the Middle East and certain areas of the African sub-continent [1]. Date palm is a perennial, monocotyledon (2*n* = 36), dioecious, cross-pollinated tree that has been widely cultivated and domestically grown in semi-arid environments since ancient times [2], [3], [4]. Depending upon the variety, a tree usually takes 4 to 6 years to become sufficiently mature to produce fruit [5]. It has been estimated that there are approximately 3,000 different cultivars of data palm, and approximately 60 are commercially cultivated and traded in the international market [6]. The fruit of these varieties vary in shape, color, size, weight and taste. Morphological variations, which are heavily dependent on environmental factors and data variety, do exist among cultivars. These variations are reflected in the diversity of the chloroplast genome, as well. For example, Zehdi et al. [7] recently showed that chloroplast diversity is 70% in eastern Algeria, while the proportion of haplotypes were lower (11 to 42%) in Egypt, Tunisia and Morocco. Additionally, the nuclear and chloroplast sequence diversity across Algeria, Morocco, Tunisia and Egypt remains unexplored [1], which can be attributed to the lack of complete chloroplast genomic information.

The chloroplast is a metabolic epicenter for maintaining plant growth and development through the photosynthesis process[8]. The genome of the chloroplast (cp) encodes numerous essential proteins for photosynthesis and metabolic processes [9, 10]. The cp genome is also used for plant systematics and taxonomy^11^. In addition, this genome acts as a source of molecular markers to perform phylogenetics due to the lower level of recombination compared to the nuclear genome [11–15]. Currently, more than 850 chloroplast genomes have been sequenced, including more than 320 chloroplast genomes from crops and trees [16]. The composition and sequence of cp genomes show significant variations within and among species [17]. Understanding the cp genome can help elucidate genomic interactions among related species for conservation and to improve valuable features of crop species[18]. Recent reports suggest that the cp genome can be used to resolve the phylogenetics of species and help in understanding genetic diversity and population dynamics [17–20].

The cp genome is comprised of a conserved quadripartite structure, which consists of a large single-copy region, a small single-copy region, and a set of inverted repeats [16, 17, 20]. Recent developments in genome sequencing technologies have allowed researchers to efficiently utilize the cp genomics data set for designing molecular barcodes and markers for detailed taxonomical systematics and phylogenetics [21]. Previously, a partial date palm genome was reported to be 380 million bp with more than 25,000 gene models [22–26], including the cp genome composition and architecture of date palm cultivars Aseel and Khalas from Pakistan and Saudi Arabia, respectively. In the case of *Arecacea* as a whole, the NCBI genome database shows a total of 3 draft genomes and 34 organelle genomes. However, the detailed gene structures and comparative taxonomic differentiation of date palm is poorly explored and reported. In the current study, we aimed to sequence the complete chloroplast genomes of two date palm cultivars ‘Khanezi’ and ‘Naghal’ to better understand the genome architecture and to compare them with available date palm cultivars (Aseel and Khalas) and related species from *Arecacea*.

## Materials and methods

### Genome sequencing and assembly

The chloroplast (cp) DNA was extracted according to the protocol of Shi *et al*. [27] with several modifications, as described by Al-Dous *et al*.[22]. We carried out complete chloroplast genome sequencing of date palm (*P*. *dactylifera* L.) cv. ‘Khanezi’ and ‘Naghal’ using the Illumina HiSeq4000 sequencing platform at Duke University, USA. A total of 26,363,570,180 and 24,702,016,614 bp were generated for Khanezi and Naghal, respectively. The raw reads were later trimmed and filtered using CLC Genomics Workbench v7.0 (CLC Bio, Aarhus, Denmark), which was also used for preparing the *de novo* genome assembly. Reads were filtered using Trimmomatic 0.36. Leading and trailing nucleotides with a phred score lower than 20 or when the phred score dropped below 20 over a 4 bp sliding window were trimmed. Illumina adapters were clipped using TruSeq 4 adapter sequences. After quality filtering and adapter trimming, reads less than 50 bp were discarded. The first assembly was made with SPADESv3.9.0, with an additional switchover to SOAPdenovo v2.04, which built assemblies from every odd K-mer from 21 to 63 bp. Contiguity and the scaffold N50 of the assembly maximized at was K = 51.

Chloroplast genomes were assembled using NCBI references from pool of assemblies using a combination of MIRA v4.0 and mitobim v1.8. Reference assemblies were assembled via 8–10 iterations with mitobim. The resulting assembly was later compared with the previously reported date palm cp genomes. Primers were designed and prepared via Macrogen Inc., South Korea to perform PCR amplification and sanger sequencing to fill gaps as in a previous report[28]. After adding the results of Sanger sequencing, the completed cp genome was used as a reference to map the initial short reads to refine the assembly based on maximum sequence coverage.

### Genome annotation and sequence architecture

A program (DOGMA) was used to annotate the Khanezi and Naghal cp genomes[29]. After the annotation, the results were compared and checked manually. Any errors in codon position were adjusted by comparing to homologs in the cp genome from NCBI. Transfer RNAs (tRNAs) were validated using tRNAscan-SE version 1.21[30] choosing the default setup. OGDRAW[8] was utilized to reveal structural features of the Khanezi and Naghal cp genomes. Relative synonymous codon usage (RSCU) was determined using MEGA7.0[31] to elucidate the divergence in synonymous codons while avoiding the influence of related amino acids. mVISTA software was utilized in Shuffle-LAGAN-mode to explore the variations in the whole cp genomes of Khanezi and Naghal cultivars compared to the two other cp genomes reported previously for Aseel and Khalas, using the Khanezi and Naghal annotation as a reference[32].

### Characterization of repeat sequence and SSRs

The REPuter program was used to show repeat sequences, which included reverse, palindromic, and direct repeats [33]. In this case, the following settings were used: a) Hamming-distance of 3, b) 90% or greater sequence-identity and c) repeat size of 30 bp. Phobosv3.3.12[34] was used to assess SSR in the chloroplast genome. The search parameters were sat at ≥10 for mononucleotides, ≥8 for dinucleotides, ≥4 for trinucleotides and tetranucleotides, and ≥3 for hexanucleotides and pentanucleotides. Additionally, tandem repeats in the cp genomes of Naghal and Khanezi cultivars were identified using TandemRepeatsFinder v4.1b with default parameters [35].

### Divergence among cp genome sequences and phylogenetic analysis

Whole cp genome and the 68 shared genes were analyzed to assess pairwise sequence divergence of the four date palm cultivars (Naghal, Khanezi, Aseel and Khalas). Missing, ambiguous and poorly annotated genes were re-confirmed by comparison and multiple sequence alignment using MAFFT(v7.222)[36] with the default settings. The Kimura-2-parameter method was used for calculating pairwise sequence divergence [37]. To resolve the Khanezi and Naghal phylogenetic positions within the family Areaceae, the 16 available cp genomes in NCBI database were used. Multiple alignments were done based on conserved structures and gene order in the cp genomes[37]. We used 4 different methods to make the trees: Bayesian-inference (MrBayes v3.1.2[38]), maximum parsimony (PAUP-4.0[39]), maximum-likelihood and neighbor joining (MEGA7.01[31]) according to the methods of Wu et al.[40, 41]. For Bayesian posterior probabilities (PP) in the BI analyses, the best substitution model GTR + G model was tested according to the Akaike information criterion (AIC) by jModelTest verion 2102. The Markov Chain Monto Carlo (MCMC) was run for 1,000,000 generations with 4 incrementally heated chains, starting from random trees and sampling 1 out of every 100 generations. The first 30% of trees were discarded as burn-in to estimate the value of posterior probabilities. Furthermore, parameters for the ML analysis were optimized with a BIONJ tree as the starting tree with 1000 bootstrap replicates using the Kimura 2-parameter model with gamma-distributed rate heterogeneity and invariant sites. MP was run using a heuristic search with 1000 random addition sequence replicates with the tree-bisection-reconnection (TBR) branch-swapping tree search criterion. In the second tier of phylogenies, a set of seventy shared genes from the cp genomes of the 16 *Areaceae* members were aligned in Clustal X with the default program settings and several manual adjustments to improve and preserve reading frames. The 4 previously mentioned phylogenetic inference models were utilized to build trees using 70 concatenated genes as described above and suggested in Asaf et al.[28]

## Results and discussion

### Sequencing and assembling the genomic data

The *de novo* assembly results showed the total sequences in data set of 1,493,007 sequences. In addition, the data set contained sequence data for 616,016,370 nucleotides. The sequencing coverage for Khanezi was 7826.8x and for Naghal was 7874.7x. The N50 values were 3,065 bp and 2,122 bp for Khanezi and Naghal, respectively.

### Chloroplast genomes of *P*. *dactylifera* L. cv. ‘Khanezi’ and ‘Naghal’

**Fig 1:**
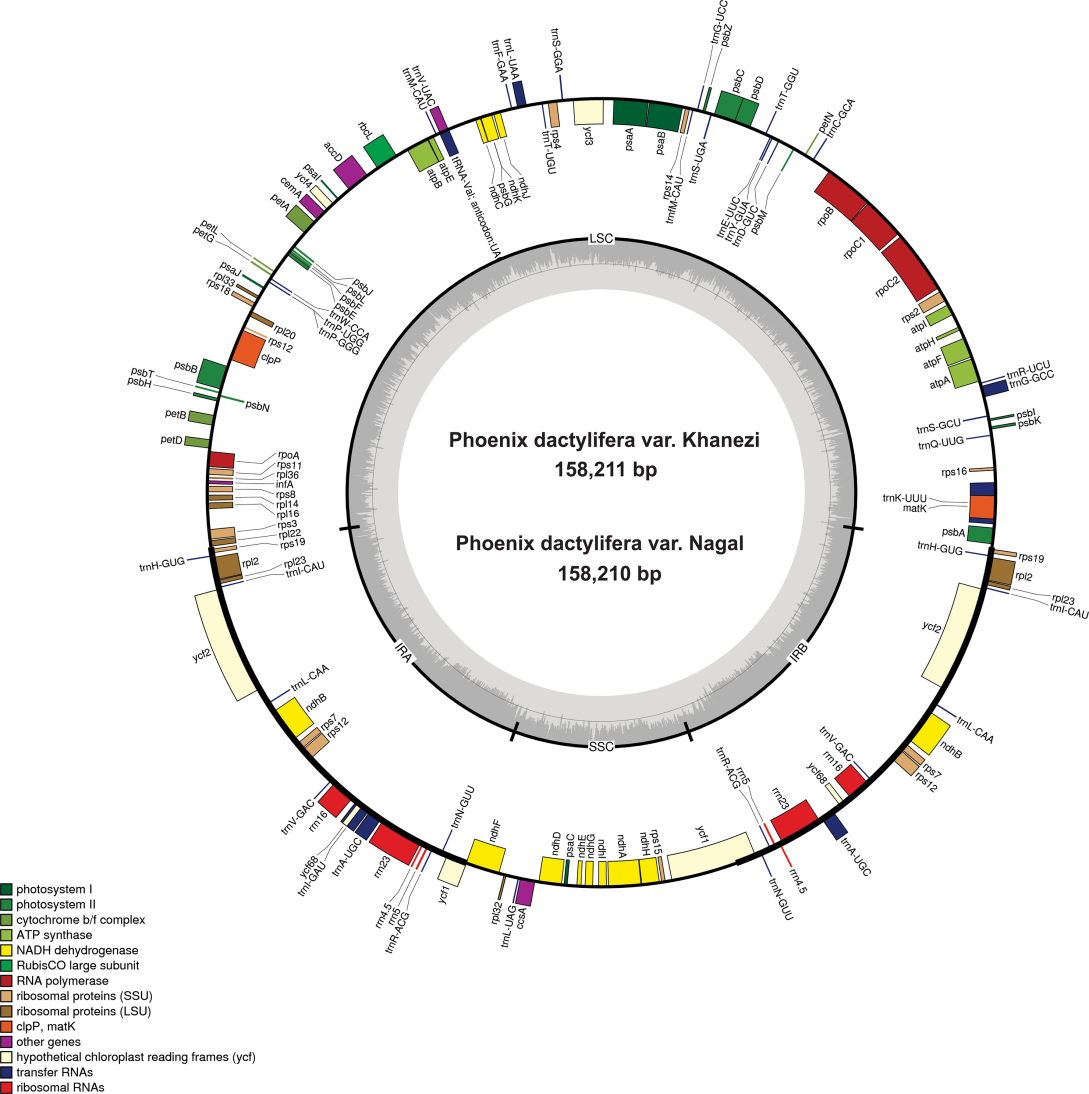
Gene map of the *P*. *dactylifera* var Khanezi and Naghal chloroplast genomes. Genes drawn inside the circle are transcribed clockwise, and those outside the circle are transcribed counter-clockwise. Asterisks indicate intron-containing genes. Genes belonging to different functional groups are color-coded. Darker gray in the inner circle corresponds to GC content, and lighter gray corresponds to AT content.

Date palm cp genomes are typical circular double-stranded DNA molecules, and they share a common quadripartite structure with the vast majority of other *Arecaceae* and angiosperms [26]. Sequence analysis and assembly revealed that Khanezi has a cp genome size of 158,211 bp, whereas the Naghal has 158,210 bp (Fig 1; Table 1). Previous cp genome analysis revealed that Khalas and Aseel have cp genome sizes of 158,462 bp and 158,458 bp, respectively [26]. This finding suggests close inter-linkage the two varieties [26]. Similar linkages and variations were also shown by Racchi et al.[42] and Khan et al.[43] for cp diversity of date palm varieties growing in Egypt, Tunisia, Morocco and Algeria. Structurally, both cp genomes in this study were comprised of four regions: a pair of inverted repeats (IR a and b), a large single copy (LSC) region and small single copy (SSC) region (Table 1; Fig 1) with varying sizes. For example, two IRs that mirrored each other showed a single bp difference in size (Khanezi—27,273 bp and Naghal—27,272 bp). In the case of LSC, the two cp genomes varied in size by two bp, i.e., 86,090 bp for Khanezi and 86,092 bp for Naghal. Similarly, the SSC was 17,575 bp and 17,574 bp for Khanezi and Naghal, respectively (Table 1). In contrast, the GC percentage was similar in the four regions. Similar patterns were also noted by Khan et al.[43] and Yang et al.[26] in Aseel and Khalas, respectively, suggesting similar GC content and differences of one to four bp across the four regions of the cp genomes (Table 1).

**Table 1:** Summary of complete chloroplast genomes for Khanezi and Naghal.

Region	*P*. *dactylifera var Khanezi*	*P*. *dactylifera var Naghal*	*P*. *dactylifera var Khalas*	*P*. *dactylifera vr Aseel*
**LSC**				
Length (bp)	86090	86092	86197	86194
GC(%)	35.3	35.3	35.3	35.3
Length (%)	54.41	54.41	54.39	54.39
**SSC**				
Length (bp)	17575	17574	17712	17711
GC(%)	31	31	30.8	30.8
Length (%)	11.10	11.10	11.17	11.17
**IR**				
Length (bp)	27273	27272	27277	27276
GC(%)	42.4	42.4	42.4	42.4
Length (%)	17.23	17.23	17.21	17.21
**Total**				
GC(%)	37.3	37.3	37.2	37.2
Length (%)	158211	158210	158462	158458

The coding sequences in both Khanezi and Naghal possess similar GC content and length relative to the cp genome; however, the length in bp was longer for Naghal than for Khanezi (Table 2). The tRNA, rRNAs and intergenic spaces were similar across the two cp genomes. However, Khalas has higher levels of rRNAs, and Aseel has lower levels of rRNAs compared to the Khanezi and Naghal cp genomes. The protein coding sequences (CDS) were 82,144 and 82,153 bp in length in Khanezi and Naghal cp genomes, respectively, and were composed of protein-coding genes contain 27,381 and 27,384 bp of codons, respectively (Table 3). Similar to other cp genomes, such as Aseel, the date palm cp genome is also AT-rich (62.7%), and the values vary slightly among non-coding, protein-coding, tRNA, and rRNA sequences, which have A+T contents of 59.5%, 62.1%, 44.7%, and 47%, respectively [26, 43]. The AT content was higher (31.1–31.7%) than GC (18.3–19.0%) in both cp genomes, and the SSC region had the highest AT and lowest GC content (Table 3). The higher AT content at the 3^rd^ position has often been used to differentiate cp DNA from nuclear and mitochondria sequences [44]. The codon utilization was estimated for tRNA and protein-coding gene sequences in both Khanezi (S1 Table) and Naghal (S2 Table) cp genomes. Most of the preferred synonymous codons (RSCU) ended with an A or U. In the cp genomes of Khanezi and Naghal, leucine (Leu; 10.2%) was the most common amino acid followed by Isoleucine and serine (8.6% and 8.1%), whereas cysteine (1.2%) was the lowest frequency amino acid (S2 and S3 Tables). Similar results were previously reported for *P*.*dactylifera* var aseel and Khalas cp genomes [26].These results also consistent with the cp genomes of other angiosperms, such as *Lonicera japonica* [45], *Oryza minuta* [46] and *Glycine max* [47].

**Table 2:** Comparison of coding and non-codign region size among *P*. *dactylifera* four varieties.

Region	*P*. *dactylifera var Khanezi*	*P*. *dactylifera var Naghal*	*P*. *dactylifera var Khalas*	*P*. *dactylifera vr Aseel*
**Protein Coding**				
Length (bp)	82144	82153	83904	81408
GC(%)	37.9	37.9	37.9	37.8
Length (%)	51.92	51.92	52.94	51.37
**tRNA**				
Length (bp)	9050	9050	9050	9050
GC(%)	55.3	55.3	55.3	55.3
Length (%)	5.7	5.7	5.71	5.71
**rRNA**				
Length (bp)	2960	2960	3568	2735
GC(%)	53	53	48.2	53.2
Length (%)	1.87	1.87	2.25	1.72
**Intergenic**				
GC(%)	40.50	40.50	39.1	41.2
Length (%)	37.3	37.3	37.2	37.3

**Table 3:** Base compositions in the Khanezi and Naghal cp genome.

	T/U		C		A		G		Length (bp)	
	Khanezi	Naghal	Khanezi	Naghal	Khanezi	Naghal	Khanezi	Naghal	Khanezi	Naghal
**Genome**	31.7	31.7	19.0	19.0	31.1	31.1	18.3	18.3	158211	158210
**LSC**	32.9	32.9	18.1	18.1	31.8	31.8	17.2	17.2	86090	86092
**SSC**	34.5	34.5	16.2	16.2	34.5	34.5	14.7	14.7	17575	17574
**IR**	28.7	28.7	20.5	20.5	28.9	28.9	28.7	28.7	27273	27272
**tRNA**	24.9	24.7	23.7	24.0	22.1	22.3	29.3	29	2960	2960
**rRNA**	18.8	18.8	23.8	23.8	25.9	25.9	31.5	31.5	9050	9050
**Protein Coding genes**	31.3	31.3	17.8	17.8	30.7	30.7	20.1	20.1	82144	82153
**1st position**	23.77	23.74	18.6	18.6	30.94	30.3	26.66	26.72	27381	27384
**2nd position**	33.50	33.53	20.4	20.3	29.38	29.4	17.72	17.8	27381	27384
**3rd position**	35.6	35.65	14.2	14.23	31.89	31.78	16.01	16.82	27381	27384

The Khanezi and Naghal cp genomes contain 111 unique genes and 19 duplicated genes in the IR. Among these unique genes, we identified 81 protein-coding, four ribosomal RNA and 29 transfer RNA genes (S4 and S5 Tables). The LSC region was comprised of sixty-two CDS and 23 t-RNA related genes. The SSC region was composed of twelve protein-coding genes and a tRNA gene. The protein-coding genes included 9 genes that encode large ribosomal proteins (*rp14*, *rp20*, *rpl2*, *rp16*, *rp23*, *rp32*, *rp22*, *rp33*, and *rp36*), twelve genes encoding small ribosomal proteins (*rps8*, *rps2*, *rps11*, *rps3*, *rps7*, *rps12*, *rps14*, *rps16*, *rps4*, *rps15*, *rps18*, and *rps19*), 5 genes encoding Photosystem-I (*psaA*, *psaB*, *psaC*, *psaI*, and *psaJ*), sixteen genes related to Photosystem-II (S6 Table), and 6 genes encoding ATP synthase and electron transport chain (*atpB*, *atpA*, *atpF*, *atpE*, *atpI* and *atpH*). Furthermore, approximately 51.92%, 5.7% and 1.87% of the cp genome sequences encoded proteins, tRNA, and rRNAs, respectively, whereas the remaining 37.4% was non-coding, including introns and intergenic spacers. The results showed that both Khanezi and Naghal cp genomes have 18 intron-containing genes, which is similar to previously reported results for the Khalas cp genome [48]. However, in the cp genome of the Aseel variety, there were 16 of these genes [43]. Among the genes that were similar to previous date palm cp genomes, almost all were single intron except for *clpP*, *ycf3* and *rps12*, the exons of which are separated by two introns (S6 Table). *Rps12* is a trans spliced gene, where an exon is in the LSC region and the other 2 reside in the IR regions separated by two introns. Similar results were reported for previously reported genomes, where the introns of all CDS shares similar splicing mechanisms as Group-II introns [49]. Among these genes, *ndhA* in SSC and *trnK-UUU* in LSC have the highest single intron size, whereas *ycf68* (replicated as well) has the lowest single intron size (S4 and S5 Tables) in the Khanezi cp genome. The *trnV-UAC* (593 bp) has a longer intron than *trnV-UAA* (513 bp). It has been shown that these kinds of introns are essential for gene regulation, and they can also affect exo-gene expression patterns depending on their specific positions. Utilizing similar introns can also increase transformational efficiency[50]. It has been observed that *ycf1, ycf2[51, 52], rpl23* [53] and *accD* [54, 55] are often absent from plants[53], but they were detected in the reported date palm cp genomes. A pair of genes (*atpB-atpE*) overlapped each other by ~4 bp. *PsbC-psbD* had a 53 bp overlap in the date palm cp genomes, whereas this overlap was 53 bp in *A*. *thaliana*, 17 bp in *A*. *arenosa*, 92 bp in *A*. *halleri* ssp *gemmifere* and *A*. *lyrata* ssp. *petraea*, 53 bp in *Gossypium* [56] and 52 bp in *Camellia* cp genomes [57]. As reported previously by Adachi *et al*. [58], there was a partial overlap of the *psbD* and ps*bC* cistrons, where translation of the *psbC* cistron is dependent on the translation of the latter *psbD* cistron. This suggests independent translation of *psbC*. Likewise, the *ndhC* and *ndhK* cistrons of the tobacco chloroplast genome also overlap, and translation of *ndhK* is strictly dependent on the upstream termination codon[59].

### Simple sequence repeat (SSR) in Naghal and Khanezi

**Fig 2:**
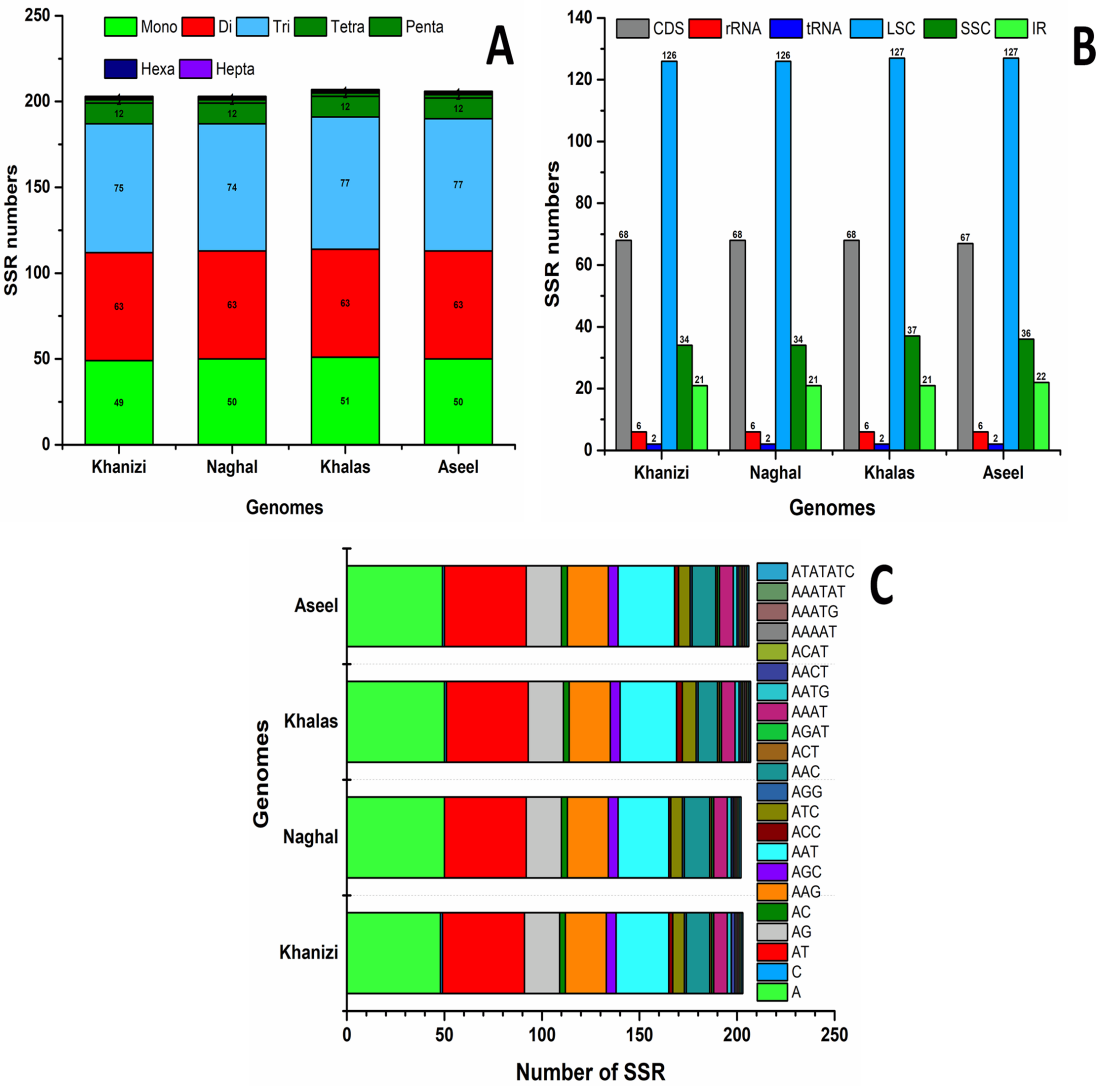
Analysis of simple sequence repeats (SSR) in the *P*. *dactylifera* var Khanezi and Naghal cp genomes. A: Number of different SSR types detected in the four genomes; B: Frequency of identified SSRs in coding, non-coding, LSC, SSC and IR regions; C: Frequency of identified SSR motifs in different repeat class types.

We analyzed the SSRs in the cp genomes of Khanezi and Naghal. During analysis, SSRs that were 10 bp or longer were defined as possible slipped strand mis-pairing due to mutational polymorphisms. From our SSR analysis, 227 and 229 microsatellites were found in the Khanezi and Naghal cp genomes, respectively (Fig 2). In Khanezi and Naghal, most mononucleotide SSRs were A motif (96.3% and 96.6%, respectively), with most SSR dinucleotides being T/A (69.54%, 71.06%) or G/A (26.31%, 27.77%) motifs (S7 Table). The chloroplast genome of Khanezi, similar to other species, contains different types of repeats that each have a specific function. The complete genome contains a different number of base pairs of the repeated sequence. Generally, as shown in S7 and S8 Tables, the whole genome more tri-base in the repeated sequence. However, there are 63 di-base pairs in the repeated sequence and 49 of mono-base pairs. The LSC region has the highest number of mono- and di-base pairs of the repeats, approximately 36 and 42, whereas Khalaas and Aseel are slightly higher at the mono level but lower at di level compared to Khanezi (S8 Table). For tri base pairs, the CDS region has a high frequency (37%) that is greater than Aseel and Khalas.

We compared perfect SSRs in Khanezi and Naghal with cp genomes of two other Aseel and Khalas cultivars. SSR has been shown to have a higher rate of mutation compared to other neutral DNA regions because of slipped DNA strands [58]. SSRs with the highest genetic diversity occur in the cp genome and are known markers used for evolutionary, population genetics, and systematics studies [60]. In the current study, SSRs measuring 10 bp or longer were found and shown to be slipped strands or mis-paired. It has been shown that mutations can be a mechanism for SSR polymorphisms [61, 62]. In Khanezi and Naghal cp genomes, we found 227 and 229 microsatellites, respectively. The current results are consistent with previous reports, where the SSR are dominated by ‘A’ or ‘T’ mononucleotide repeats[63, 64]. These different kinds of SSR repeats (mononucleotide, pentanucleotide, and hexanucleotides) are comprised of A or T bases at higher frequencies, which corresponds to the biased-base composition and A/T richness of the cp genomes[65, 66]. These results are consistent with previous reports that show that the SSRs in cp genome contain polythymine (polyT) or polyadenine (polyA) repeats in addition to infrequent tandem cytosine and guanine repeats[66]. Therefore, the existence of such SSRs in the cp genome considerably contributes to the ‘AT’ ratio shown for the Khanezi and Naghal genomes. This phenomenon was also previously reported for different species [67, 68]. The current findings suggest that approximately 69% (Khanezi) and 77% (Naghal) SSRs were detected in non-coding regions. These results are consistent with previous studies determined that SSRs as unequally distributed in the chloroplast. In addition, these data might also provide information for designing targeted markers for detecting intra- and interspecific polymorphisms for date palm cultivars [69, 70].

### Repeat sequence and comparative distribution in date palm

**Fig 3:**
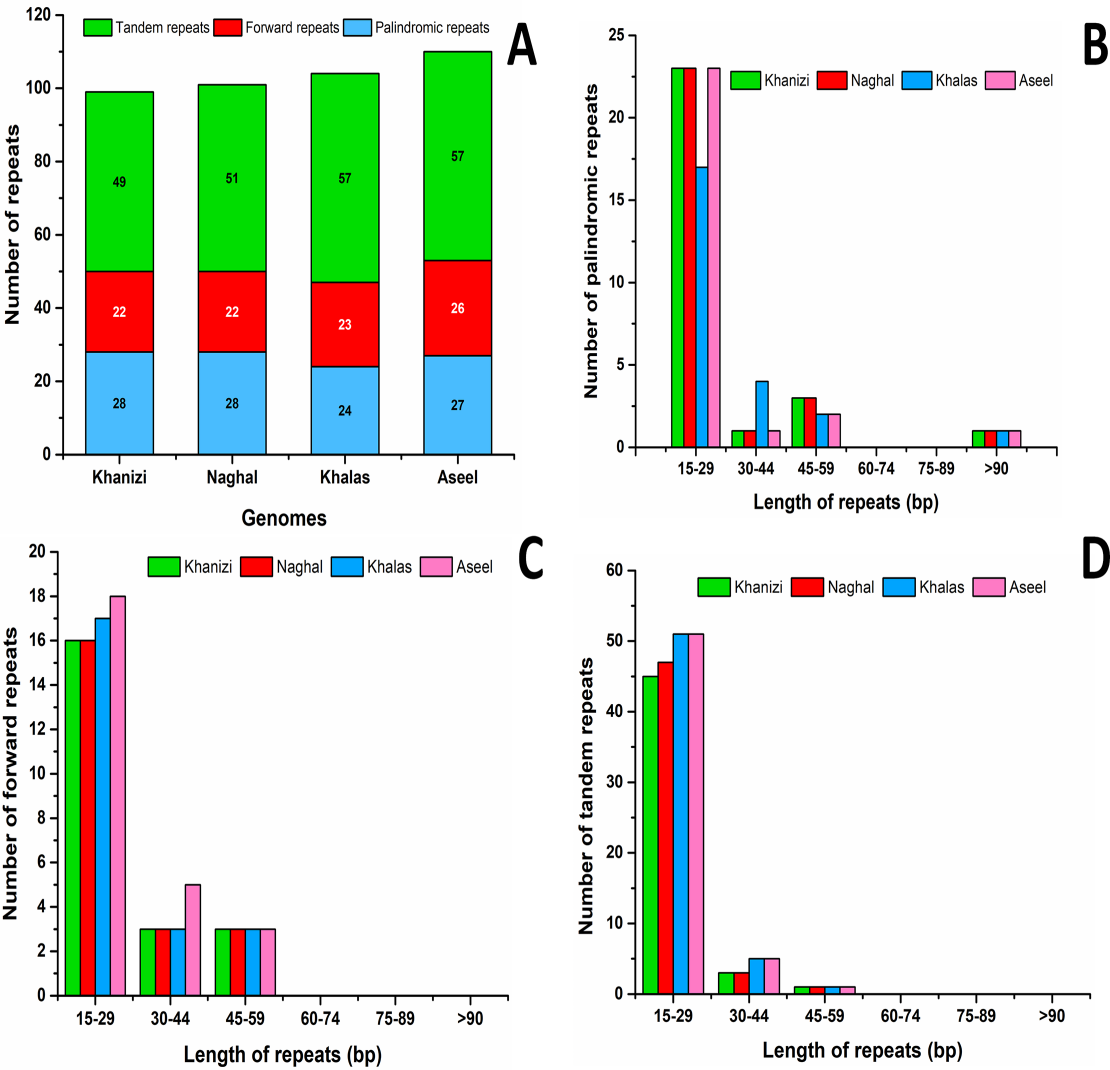
Analysis of repeated sequences in *P*. *dactylifera* var Khanezi and Naghal cp genomes. A: Totals of three repeat types; B: Frequency of palindromic repeats by length; C: Frequency of forward repeats by length; D: Frequency of tandem repeats by length.

The results showed that 99 and 101 repeats were found in the cp genomes of Khanezi and Naghal, respectively, which included 28 palindromic, 22 direct and 49 and 51 palindromic repeats (Fig 3). Among these repeats, 23 palindromic repeats were 15–29 bp in length, whereas there was one repeat each 30–44 bp and >90 bp in length and 3 palindromic repeats 45–59 bp in length. Another type of repeated sequence, forward repeats, occurred in these cp genomes at different frequencies. For example, there were 16 15–29 bp forward repeats, whereas there were 3 forward repeats, with each measuring 30–44 and 45–59 bp in length. In addition to these repeats, tandem repeats occurred in high numbers. Tandem repeats 15–29 bp in length were identified with frequency of 45 and 47 in the Naghal and Khanezi cp genomes, respectively. Similarly, 30–44 and 45–59 bp tandem repeats were found at a frequency of three and one, respectively (Fig 3). In comparison to both Naghal and Khanezi, the most tandem repeats were found in the cp genomes of the Aseel and Khalas varieties, with a frequency of 57. Repeat sequences are very helpful in phylogenetic studies and play a role in genome rearrangements[71, 72]. Analyses of various chloroplast genomes concluded that repeats are important in inducing indels and substitutions[73]. The length of direct and palindromic repeats in the Khanezi and Naghal cp genomes were considerably short ranging from 30–101 bp. In this case, similar results were previously shown for the cp genome of *Camellia* species, which have eighty-two repeats. In contrast, other reports have shown longer repeats of 132 bp and 287 bp in *Poaceae* and *Fabaceae*, respectively[74]. Recent studies have shown that variations in sequence and rearrangement of genomes can be due to slipped-strand un/mispairing and improper re-combination of repeats[75, 76]. Additionally, the occurrence of these repeats suggests that the regions are important hotspots for reconfiguration of the cp genome [76]. The data related to repeats could be utilized to develop molecular markers for understanding the population dynamics of Khanezi and Naghal [71].

### Structural comparison of date palm cp genomes

**Fig 4:**
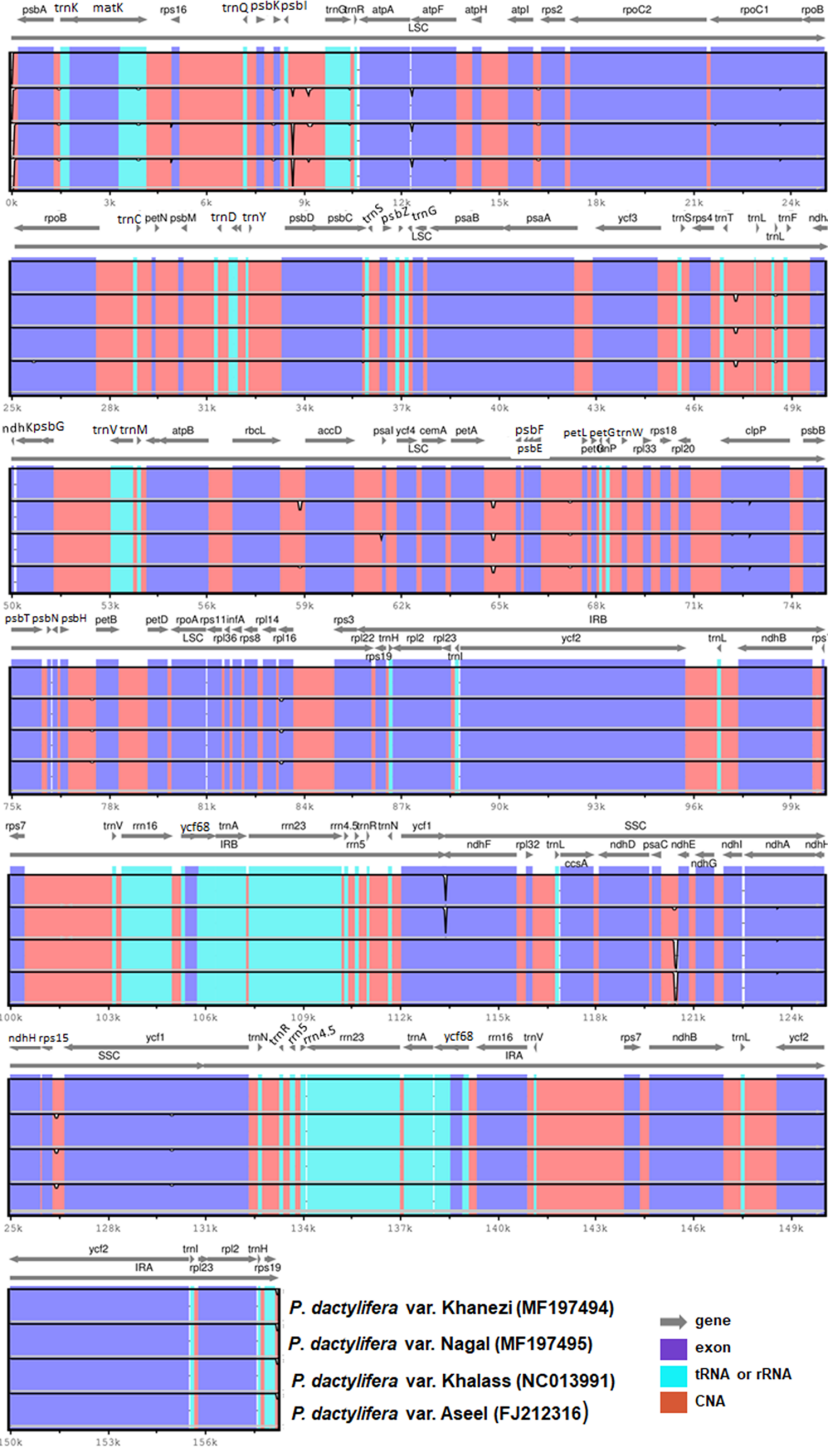
Alignment visualization of the *P*. *dactylifera* var Khanezi and Naghal chloroplast genome sequences. VISTA-based identity plot showing sequence identity among the four-species using *P*. *dactylifera* var Khalas as a reference. The vertical scale indicates percent identity, ranging from 50% to 100%. The horizontal axis indicates the coordinates within the chloroplast genome. Arrows indicate the annotated genes and their transcription direction. The thick black lines show the inverted repeats (IRs).

The date palm tree cp genomes evolve slowly, where the total rate of nucleotide substitution is approximately eightfold lower than observed in annual plants. Because previous cp genome studies were published almost five years ago with little focus on the comparative assessment among cp genomes of date palm cultivars, we analyzed two previously reported complete cp genomes from Aseel and Khalas cultivars together with the Naghal and Khanezi genomes from the current study. Among these cp genomes, Naghal was the smallest (158,210 bp), whereas Khalas had the largest cp genome size (158,462 bp). In addition, the difference in length between Naghal and Khanezi was a single base pair, whereas Khalas and Aseel had a 4 bp difference. Pairwise cp genomic alignment of these four cp genomes uncovered a high degree of synteny. Using the mVISTA algorithm, the sequences of the four available date palm cp genomes were compared (Fig 4).

The results showed comparatively low sequence identity among the cp genomes of the four varieties, especially in *atpF*, *rpoC1*, *clpP*, *rpl16*, *ndhA*, *ycf1* and *ndhF*. Similar to previous reports on various cp genomes [26, 43], Naghal and Khanezi cp genomes also showed more divergence in the LSC, SSC and non-coding regions and compared to the IR and coding regions, respectively. Among the non-coding sequences, highly divergent regions included *psbK-trnG*, *trnT-trnL*, *rbcL-accD*, *petA-psbJ* and *psaC-ndhE* spacers as reported previously. In addition, previous studies have shown that coding and non-coding areas with high variation, such as *trnS(GGA*)-*trnG(UCC)*, *rpl16-rps3*, *trnT-trnL* and *atpB-rbcL*, have led to the development of potential genetic markers in angiosperms. Furthermore, comparison of Khanezi and Naghal cp genomes with related varieties revealed various useful results, including that Khanezi showed 35 indels and 23 SNPs with Naghal. In contrast, Aseel and Khalaas have more *indels*, 293 and 299, respectively with Khanizi. In contrast, the number of SNPs in Aseel and Khalaas are 18 and 16. Similarly, Naghal revealed 292 and 296 indels and 10 and 12 SNPs in Aseel and Khalas, respectively (S9 Table). We further compared the Khanezi and Naghal cp genomes and calculated the average pairwise sequence divergence among the four varieties (S10 Table). Khanezi and Naghal exhibited 0.000120 and 0.000192 average sequence divergence, respectively. Khanezi showed more divergence from Khalas and Aseel (0.00108 and 0.000101, respectively) compared to the divergence of Naghal from these two varieties (0.000071 and 0.000076, respectively).

**Fig 5:**
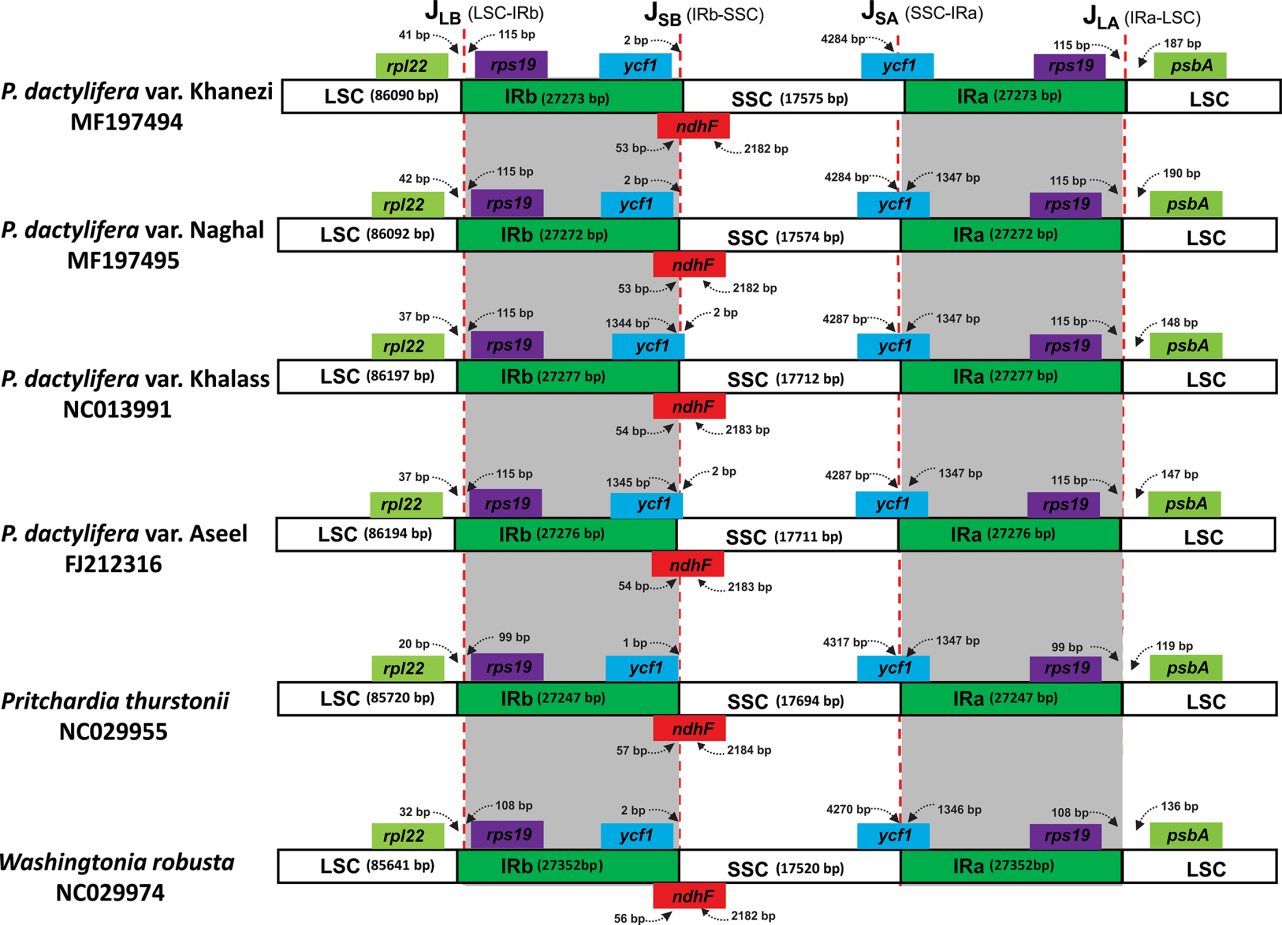
Comparison of border distance between adjacent genes and junctions of the LSC, SSC, and two IR regions among the chloroplast genomes of *P*. *dactylifera* var Khanezi and Naghal. Boxes above or below the main line indicate the adjacent border genes. The figure is not to scale with respect to sequence length and only shows relative changes at or near the IR/SC borders.

In the case of IRs, the contraction and expansion of the border regions have been posited as main features of cp genome size variation and have also been credited for evolution[77–79]. Considerable expansion and contraction of the IR region is mostly responsible for the size variation observed among chloroplast genomes [13, 80]. In this study, we compared the position of IR borders of four date palm varieties with two *Arecaceae* members *Pritchardia thurstonii* and *Washingtonia robusta*. Due to a characteristic expansion of IRB sequences into the LSC region, a specific rearrangement was acquired by monocot cp genomes early in their evolution. This expansion resulted in the inclusion of *trnH* and *rps19* genes in the IR region. The distance between J_LB_ and *rps19* is 115 bp in all date palm verities, and is observed to be 99 bp and 108 bp in *P*. *thurstonii* and *W*. *robusta*, respectively (Fig 5). Similarly, J_LA_ is located between *rps19* and *psbA*, and the distance between *psbA* and J_LA_ ranges from 147 to 187 bp among the four varieties. In Naghal and Khaneizi, this distance was 190 bp and 187 bp, respectively. However, in *P*. *thurstonii* and *W*. *robusta*, this distance was 119 bp and 136 bp, respectively. Similar results were obverted by Yang et al.[26] and Khan et al.[43] for the Khalas and Aseel cp genomes.

### Phylogenetic analysis of *P*. *dactylifera* var Khanezi and Naghal

**Fig 6:**
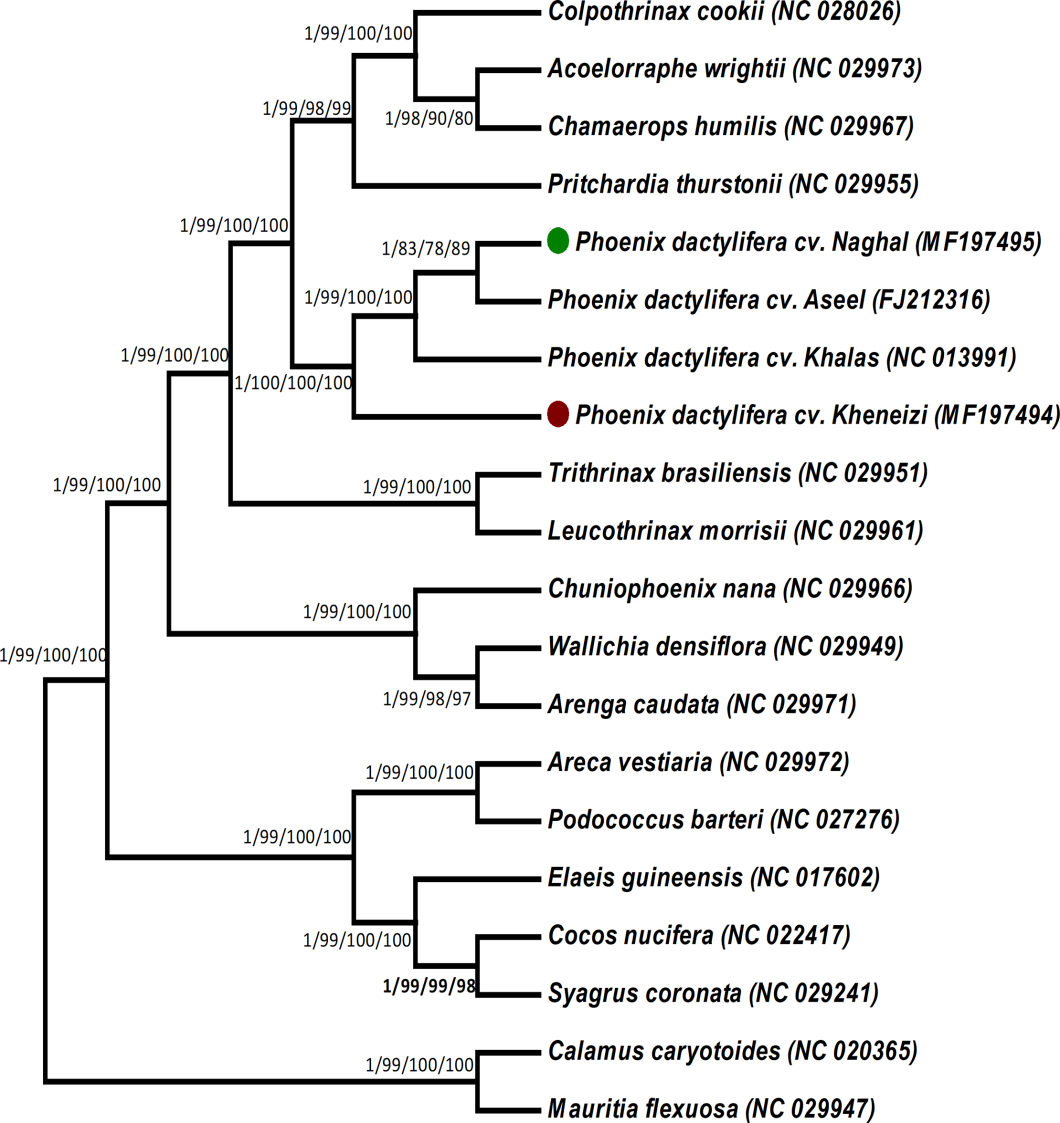
Phylogenetic trees were constructed for twenty species from the family Areaceae using several different methods, and the tree shown is for the 68 shared protein-coding genes. The following four methods were used for the 68 shared genes data set: Bayesian inference (BI), maximum parsimony (MP), maximum likelihood (ML) and neighbor-joining (NJ). Numbers above the branches are the posterior probabilities of BI and bootstrap values for NJ, MP and ML. Green and brown dots represent the positions of *P*. *dactylifera* var Khanezi and Nagha.

The phylogenetic position of *P*. *dactylifera* var Khanezi and Naghal within the family *Arecaeae* was established by analyzing multiple alignments of complete cp genomes and 68 shared genes of 16 *Arecaeae* members, representing seventeen genera (Fig 6 and S1 Fig). Phylogenetic analyses using maximum likelihood, Bayesian-inference, maximum-parsimony, and neighbor joining were performed. The results revealed that the complete cp genomes and 68 shared genes of *P*. *dactylifera* var Khanezi and Naghal contain the same phylogenetic signals; the complete genome sequence and the 68 shared genes (from all species) generated phylogenetic trees with identical topologies (Fig 6 and S1 Fig). In these phylogenetic trees based on the entire genome data set and the 68 shared genes, *P*. *dactylifera* var Khanezi and Naghal formed a single clade with Khalas with high Bayesian inference and bootstrap support using 4 phylogeny models (Fig 6 and S1 Fig). The results revealed that Naghal is closer to Aseel compared to Khalas and Khanezi. Most of the previous studies concerning the phylogenetic analysis of date palm cultivar used SSR, RAPD (random amplified polymorphic DNA) and DAMD (directed amplification of minisatellites DNA) markers to understand the genetic discrimination [81–83]. Akhtar et al. [84] showed *rps14* for understanding the phylogenetic relationship among Pathri, Dhaddy, Makhi, Aseel, and Khudrawi date palm cultivars from Pakistan. Similarly, specific SSRs were used to differentiate among Khalas, Hillali, Khnaizi, and Jabri from Qatari date palm cultivars [85]. However, the current study for the first time used four different phylogenetic approaches and 68 shared genes to construct the phylogeny, suggesting a clear differentiation of the four cultivars.

## Conclusions

This study produced the first complete chloroplast genome of two important cultivars (Naghal and Khanezi) growing in the Sulatanate of Oman and the rest of the Arabian Peninsula. The genomic data were assembled and analyzed, and the genomes were compared with the only two other reported cultivars of date palm (Aseel and Khalas). Genome arrangements, gene content and order, and codon usage were consistent with the previously elucidated cp genomes from the genus *Phoenix*. The location and distribution of repeat sequences was determined, and sequence divergence of cp genomes and 68 shared genes were calculated for related species. The phylogenetic analysis based on whole cp genomes and 68 shared genes yielded identical phylogenetic trees, with Khanezi and Naghal forming single clades with Khalas and Aseel cultivars, respectively.

## Supporting information

S1 Table Codon–anticodon recognition pattern and codon usage for the Khanezi chloroplast genome. (DOCX)

S2 Table Codon–anticodon recognition pattern and codon usage for the Khanezi chloroplast genome. (DOCX)

S3 Table Amino acid frequencies and percentages in Khanezi and Naghal cp genomes. (DOCX)

S4 Table Genes with introns in the Naghal chloroplast genome and the length of exons and introns. (DOCX)

S5 Table Genes with introns in the Khanezi chloroplast genome and the length of exons and introns. (DOCX)

S6 Table Genes in the sequenced Khanezi and Naghal chloroplast genomes. (DOCX)

S7 Table Simple sequence repeats (SSRs) in the Naghal chloroplast genome. (DOCX)

S8 Table Simple sequence repeats (SSRs) in the Khanezi chloroplast genome. (DOCX)

S9 Table Indel and SNP analysis of cp genomes from Khanezi an Naghal with other two date palm varieties. (DOCX)

S10 Table Average pairwise distance of cp sequences from Khanezi and Naghal with other two varieties. (XLS)

S1 Fig Phylogenetic trees were constructed for twenty species from the family Areaceae using several different methods, and the tree shown is for the entire genome sequence. The following four methods were used for the entire genome data set: Bayesian inference (BI), maximum parsimony (MP), maximum likelihood (ML) and neighbor-joining (NJ). Numbers above the branches are the posterior probabilities of BI and bootstrap values for NJ, MP and ML. Green and brown dots represent the positions of *P*. *dactylifera* var Khanezi and Nagha.
(TIF)
