# Supplementary material for: First chloroplast genomics study of Phoenix dactylifera (var. Naghal and Khanezi): A comparative analysis
Source: PLoS One. 2018 Jul 31;13(7):e0200104. doi: 10.1371/journal.pone.0200104 (PMC6067692; doi:10.1371/journal.pone.0200104)
Supplement: S1 Table — (DOCX) [file pone.0200104.s001.docx]

**Table S1**. The codon–anticodon recognition pattern and codon usage for the Naghal chloroplast genome**.**

| **Amino acid** | **Codon** | **No** | **RSCU** | **tRNA** | **Amino acid** | **Codon** | **No** | **RSCU** | **tRNA** |
| --- | --- | --- | --- | --- | --- | --- | --- | --- | --- |
| Phe | UUU | 974 | 1.24 |  | Ala | GCA | 389 | 1.18 | *trnA-UGC* |
| Phe | UUC | 590 | 0.75 | *trnF-GAA* | Ala | GCG | 145 | 0.5 |  |
| Leu | UUA | 839 | 1.9 | *trnL-UAA tRNA* | Tyr | UAU | 833 | 1.5 |  |
| Leu | UUG | 590 | 1.1 | *trnL-CAA tRNA* | Tyr | UAC | 212 | 0.47 | *trnY-GUA tRNA* |
| Leu | CUU | 576 | 1.29 |  | Stop | UAG | 1 | 0.74 |  |
| Leu | CUC | 215 | 0.4 |  | Stop | UGA | 0 | 0.80 |  |
| Leu | CUA | 388 | 0.87 | *trnL-UAG tRNA* | Stop | UAA | 2 | 1.44 |  |
| Leu | CUG | 186 | 0.32 |  | His | CAU | 508 | 1.49 |  |
| Ile | AUU | 1109 | 1.51 |  | His | CAC | 158 | 0.50 | *trnH-GUG tRNA* |
| Ile | AUC | 508 | 0.5 | *trnI-GAU tRNA* | Gln | CAA | 712 | 1.53 | *trnQ-UUG tRNA* |
| Ile | AUA | 738 | 0.89 |  | Gln | CAG | 246 | 0.49 |  |
| Met | AUG | 651 | 1 | *trnM-CAU tRNA* | Asn | AAU | 1033 | 1.44 |  |
| Val | GUU | 531 | 1.50 |  | Asn | AAC | 287 | 0.55 | *trnQ-UUG tRNA* |
| Val | GUC | 195 | 0.46 | *trnV-GAC tRNA* | Lys | AAA | 1064 | 1.44 | *trnK-UUU tRNA* |
| Val | GUA | 564 | 1.47 | *trnV-UAC tRNA* | Lys | AAG | 385 | 0.55 |  |
| Val | GUG | 215 | 0.54 |  | Asp | GAU | 907 | 1.55 |  |
| Ser | UCU | 619 | 1.56 |  | Asp | GAC | 225 | 0.44 | *trnD-GUC tRNA* |
| Ser | UCC | 372 | 1.23 | *trnS-GGA tRNA* | Glu | GAA | 1100 | 1.48 | *trnE-UUC tRNA* |
| Ser | UCA | 469 | 1.03 | *trnS-UGA tRNA* | Glu | GAG | 376 | 0.51 |  |
| Ser | UCG | 211 | 0.48 |  | Cys | UGU | 256 | 1.50 |  |
| Ser | AGU | 425 | 1.24 |  | Cys | UGC | 82 | 0.49 |  |
| Ser | AGC | 111 | 0.42 | *trnS-GCU tRNA* | Trp | UGG | 458 | 1 | *trnW-CCA tRNA* |
| Pro | CCU | 438 | 1.59 |  | Arg | CGU | 369 | 1.36 | *trnR-ACG tRNA* |
| Pro | CCC | 221 | 0.86 |  | Arg | CGC | 92 | 0.51 |  |
| Pro | CCA | 341 | 1.07 | *trnP-UGG tRNA* | Arg | CGA | 371 | 1.24 |  |
| Pro | CCG | 134 | 0.47 |  | Arg | CGG | 144 | 0.48 |  |
| Thr | ACU | 552 | 1.68 |  | Arg | AGA | 546 | 1.77 | *trnR-UCU tRNA* |
| Thr | ACC | 259 | 0.76 | *trnT-GGU tRNA* | Arg | AGG | 176 | 0.61 |  |
| Thr | ACA | 459 | 1.08 | *trnT-UGU tRNA* | Gly | GGU | 614 | 1.28 |  |
| Thr | ACG | 164 | 0.45 |  | Gly | GGC | 157 | 0.42 |  |
| Ala | GCU | 617 | 1.72 |  | Gly | GGA | 750 | 1.52 | *trnG-UCC tRNA* |
| Ala | GCC | 214 | 0.59 |  | Gly | GGG | 303 | 0.77 |  |
