# Supplementary material for: First chloroplast genomics study of Phoenix dactylifera (var. Naghal and Khanezi): A comparative analysis
Source: PLoS One. 2018 Jul 31;13(7):e0200104. doi: 10.1371/journal.pone.0200104 (PMC6067692; doi:10.1371/journal.pone.0200104)
Supplement: S3 Table — (DOCX) [file pone.0200104.s003.docx]

**S3 Table. Amino acid frequencies and percentages in Khanezi and Naghal cp genomes**

| **Amino acid** | **Khanezi** | | **Naghal** | | **Aseel** | | **khalas** | |
| --- | --- | --- | --- | --- | --- | --- | --- | --- |
|  | **Frequency** | **percentage** | **Frequency** | **percentage** | **Frequency** | **percentage** | **Frequency** | **percentage** |
| **Ala** | 1365 | 5 | 1365 | 5 | 1350 | 5 | 1423 | 5 |
| **Cys** | 338 | 1.2 | 338 | 1.2 | 333 | 1.2 | 344 | 1.2 |
| **Asp** | 1132 | 4.1 | 1132 | 4.1 | 1115 | 4.1 | 1150 | 4.1 |
| **Glu** | 1475 | 5.4 | 1476 | 5.4 | 1462 | 5.4 | 1506 | 5.3 |
| **Phe** | 1564 | 5.7 | 1564 | 5.7 | 1558 | 5.7 | 1606 | 5.7 |
| **Gly** | 1824 | 6.7 | 1824 | 6.7 | 1802 | 6.6 | 1908 | 6.8 |
| **His** | 666 | 2.4 | 666 | 2.4 | 660 | 2.4 | 676 | 2.4 |
| **Ile** | 2355 | 8.6 | 2355 | 8.6 | 2341 | 8.6 | 2418 | 8.6 |
| **Lys** | 1451 | 5.3 | 1449 | 5.3 | 1422 | 5.2 | 1484 | 5.3 |
| **Leu** | 2792 | 10.2 | 2794 | 10.2 | 1770 | 10.2 | 2862 | 10.1 |
| **Met** | 659 | 2.4 | 659 | 2.4 | 658 | 2.4 | 681 | 2.4 |
| **Asn** | 1320 | 4.8 | 1320 | 4.8 | 1309 | 4.8 | 1339 | 4.7 |
| **Pro** | 1134 | 4.1 | 1134 | 4.1 | 1113 | 4.1 | 1179 | 4.2 |
| **Gln** | 956 | 3.5 | 958 | 3.5 | 945 | 3.5 | 981 | 3.5 |
| **Arg** | 1698 | 6.2 | 1698 | 6.2 | 1670 | 6.2 | 1770 | 6.3 |
| **Ser** | 2211 | 8.1 | 2207 | 8.1 | 2173 | 8 | 2225 | 7.9 |
| **Thr** | 1432 | 5.2 | 1434 | 5.2 | 1407 | 5.2 | 1475 | 5.2 |
| **Val** | 1504 | 5.5 | 1505 | 5.5 | 1476 | 5.4 | 1579 | 5.6 |
| **Trp** | 458 | 1.7 | 458 | 1.7 | 453 | 1.7 | 471 | 1.7 |
| **Tyr** | 1044 | 3.8 | 1045 | 3.8 | 1030 | 3.8 | 1074 | 3.8 |
| **Stop** | 83 | 0.3 | 86 | 0.3 | 89 | 0.3 | 95 | 0.3 |
