# Supplementary material for: First chloroplast genomics study of Phoenix dactylifera (var. Naghal and Khanezi): A comparative analysis
Source: PLoS One. 2018 Jul 31;13(7):e0200104. doi: 10.1371/journal.pone.0200104 (PMC6067692; doi:10.1371/journal.pone.0200104)
Supplement: S4 Table — (DOCX) [file pone.0200104.s004.docx]

**S4 Table.** **The genes with introns in the Naghal chloroplast genome and the length of exons and introns**

| **Gene** | **Location** | **Exon I (bp)** | **Intron 1 (bp)** | **Exon II (bp)** | **Intron II (bp)** | **Exon III (bp)** |
| --- | --- | --- | --- | --- | --- | --- |
| *atpF* | LSC | 144 | 783 | 408 |  |  |
| *clpP* | LSC | 69 | 820 | 291 | 647 | 249 |
| *ndhA* | SSC | 552 | 1041 | 537 |  |  |
| *ndhB^a^* | IR | 777 | 709 | 753 |  |  |
| *rpl2^a^* | IR | 387 | 659 | 429 |  |  |
| *rpoC1* | LSC | 435 | 727 | 1623 |  |  |
| *rps12** |  | 114 | - | 29 | 541 | 229 |
| *ycf3* | LSC | 132 | 714 | 228 | 732 | 156 |
| *ycf68^a^* |  | 42 | 28 | 303 |  |  |
| *trnA-UGC* | IR | 38 | 802 | 35 |  |  |
| *trnI -GAU* | IR | 42 | 939 | 35 |  |  |
| *trnL-UAA* | LSC | 35 | 513 | 50 |  |  |
| *trnK -UUU* | LSC | 37 | 2623 | 29 |  |  |
| *trnV-UAC* | LSC | 39 | 593 | 37 |  |  |
| *trnG-GCC* | LSC | 35 | 647 | 37 |  |  |

*^a^ Genes replicated:*

*the rps12 gene is divided into 5`-rps12 in the LSC region and 3`-rps12 in the IR region.
