# Supplementary material for: First chloroplast genomics study of Phoenix dactylifera (var. Naghal and Khanezi): A comparative analysis
Source: PLoS One. 2018 Jul 31;13(7):e0200104. doi: 10.1371/journal.pone.0200104 (PMC6067692; doi:10.1371/journal.pone.0200104)
Supplement: S7 Table — (DOCX) [file pone.0200104.s007.docx]

**S7 Table.** Simple sequence repeats (SSRs) in the Naghal chloroplast genome

| **Unit** | **Length** | **No** | **SSR start** |
| --- | --- | --- | --- |
| **A** | 16 | 2 | 4897, 9166 |
|  | 14 | 1 | 82623 |
|  | 13 | 1 | 125970 |
|  | 12 | 2 | 3902, 19230 |
|  | 11 | 16 | 33, 8110, 12252, 19125, 29499, 29772, 33181, 37557, 51913, 67183, 84693, 86089, 123586, 127490, 127613, 129917 |
|  | 10 | 28 | 3875, 9092, 9331, 13052, 13096, 13780, 14469, 14530, 33030, 46811, 48487, 51466, 58867, 60583, 60792, 62633, 68726, 72188, 72458, 72721, 81756, 113219, 115645, 116461, 118054, 124065, 130035, 131076 |
| **AT** | 15 | 2 | 14903, 61220 |
|  | 14 | 2 | 47294, 120302 |
|  | 13 | 1 | 115657 |
|  | 12 | 3 | 14936, 48200, 123408 |
|  | 11 | 3 | 47321, 51759, 69841 |
|  | 10 | 6 | 3445, 9031, 20595, 47623, 49124, 115676 |
|  | 9 | 5 | 8581, 23598, 83806, 97002, 147294 |
|  | 8 | 20 | 1387, 4185, 9011, 9104, 9125, 15187, 20505, 29621, 29639, 29652, 29660, 30003, 33109, 46648, 48915, 56599, 71729, 95451, 126677, 148846 |
|  |  |  |  |
| **AG** | 11 | 1 | 125475 |
|  | 10 | 1 | 62699 |
|  | 9 | 4 | 8389, 23625, 35899, 41646 |
|  | 8 | 12 | 48346, 88860, 88872, 89874, 97743, 109044, 129977, 135253, 146554, 154423, 155425, 155437 |
| **AC** | 8 | 3 | 6113, 29120, 60127 |
|  |  |  |  |
| **AAT** | 19 | 1 | 120379 |
|  | 13 | 1 | 83796 |
|  | 12 | 1 | 120368 |
|  | 11 | 3 | 37673, 120404, 128706 |
|  | 10 | 5 | 36658, 83751, 116751, 128689, 128725 |
|  | 9 | 15 | 8982, 14930, 27877, 37661, 47210, 54083, 61214, 64826, 69254, 80959, 83760, 114974, 115635, 120349, 126338 |
| **AGG** | 11 | 1 | 82149 |
| **AAG** | 10 | 8 | 22863, 30766, 59671, 73202, 86788, 99506, 144789, 157507 |
|  | 9 | 13 | 69409, 70388, 92757, 92869, 94620, 98038, 102770, 117507, 141526, 146258, 149676, 151427, 151539 |
| ATC | 11 | 1 | 31946 |
|  | 10 | 1 | 39164 |
|  | 9 | 4 | 93375, 93939, 150357, 150921 |
| AAC | 9 | 11 | 4342, 15425, 40769, 50706, 52607, 70135, 103572, 112498, 130427, 131798, 140724 |
| ACC | 11 | 1 | 28912 |
|  | 9 | 2 | 93133, 151163 |
| AGC | 9 | 5 | 41062, 85847, 107480, 124210, 136816 |
| ACT | 9 | 1 | 28041 |
| **AGAT** | 15 | 1 | 6149 |
| **AAAT** | 14 | 1 | 8636 |
|  | 13 | 2 | 72919, 84339 |
|  | 12 | 4 | 8618, 29626, 118313, 121016 |
| AATG | 14 | 1 | 63309 |
|  | 12 | 1 | 123516 |
| **AACT** | 12 | 1 | 16098 |
| **ACAT** | 12 | 1 | 84436 |
| **AAAAT** | 15 | 1 | 42398 |
| **AAATG** | 15 | 1 | 66748 |
| **ATATATC** | 21 | 1 | 36772 |
