# Supplementary material for: First chloroplast genomics study of Phoenix dactylifera (var. Naghal and Khanezi): A comparative analysis
Source: PLoS One. 2018 Jul 31;13(7):e0200104. doi: 10.1371/journal.pone.0200104 (PMC6067692; doi:10.1371/journal.pone.0200104)
Supplement: S8 Table — (DOCX) [file pone.0200104.s008.docx]

**Table 8. Simple sequence repeats (SSRs) in the Khanezi chloroplast genome**

| **Unit** | **Length** | **No** | **SSR start** |
| --- | --- | --- | --- |
|  |  |  |  |
| **A** | 17 | 1 | 9173 |
|  | 16 | 1 | 4895 |
|  | 14 | 1 | 82622 |
|  | 13 | 1 | 125970 |
|  | 12 | 3 | 3900, 19232, 58870 |
|  | 11 | 16 | 30, 3872, 8108, 12260, 19127, 29500, 29773, 33182, 37558, 51918, 67182, 84692, 86088, 127490, 127613, 129917 |
|  | 10 | 25 | 9096, 9339, 13054, 13098, 13782, 14471, 14532, 33031, 46812, 51471, 60588, 60797, 62638, 68725, 72456, 72719, 81755, 113218, 115644, 116460, 118053, 123587, 124065, 130035, 131076 |
| C | 13 | 1 | 58857 |
| **AT** | 18 | 1 | 9107 |
|  | 15 | 2 | 14905, 61225 |
|  | 14 | 2 | 47300, 120301 |
|  | 13 | 1 | 115656 |
|  | 12 | 3 | 14938, 48206, 123409 |
|  | 11 | 3 | 47327, 51764, 69840 |
|  | 10 | 6 | 3442, 9035, 20597, 47629, 49129 |
|  | 9 | 5 | 8579, 23600, 83805, 97001, 147294 |
|  | 8 | 19 | 1384, 4183, 9015, 9132, 15189, 20507, 29622, 29640, 29653, 29661, 30004, 33110, 46649, 48920, 56604, 71728, 95450, 126677, 148846 |
|  |  |  |  |
| **AG** | 11 | 1 | 125475 |
|  | 10 | 1 | 62704 |
|  | 9 | 4 | 8387, 23627, 35900, 41647 |
|  | 8 | 12 | 48352, 88859, 88871, 89873, 97742, 109043, 129977, 135253, 146554, 154423, 155425, 155437 |
| **AC** | 8 | 3 | 6111, 29121, 60132 |
|  |  |  |  |
| **AAT** | 19 | 1 | 120378 |
|  | 13 | 1 | 83795 |
|  | 12 | 1 | 120367 |
|  | 11 | 3 | 37673, 120404, 128706 |
|  | 10 | 5 | 36659, 83750, 116750, 128689, 128725 |
|  | 9 | 12 | 8655, 8986, 14932, 27878, 37662, 47211, 54088, 61219, 64831, 69253, 80958, 83759 |
| **AAG** | 11 | 1 | 82148 |
|  | 10 | 8 | 22865, 30767, 59676, 73200, 86787, 99505, 144789, 157507 |
|  | 9 | 13 | 69408, 70387, 92756 92868, 94619, 98037, 102769, 117506, 141526, 146258, 149676, 151427,151539 |
| ATC | 11 | 1 | 31947 |
|  | 10 | 1 | 39165 |
|  | 9 | 4 | 93374, 93938, 150357, 150921 |
| AAC | 9 | 12 | 4340, 15427, 40770, 50711, 52612, 70134, 93132, 103571, 112497, 130427, 131798, 140724 |
| ACC | 11 | 1 | 28913 |
|  | 9 | 1 | 151163 |
| AGC | 9 | 5 | 41063, 85846, 107479, 124210, 136816 |
| ACT | 9 | 1 | 28042 |
| **AGAT** | 15 | 1 | 6147 |
| **AAAT** | 14 | 1 | 8634 |
|  | 13 | 2 | 72917, 84338 |
|  | 12 | 4 | 8616, 29627, 118312, 121017 |
| AATG | 14 | 1 | 63314 |
|  | 12 | 1 | 123517 |
| **AACT** | 12 | 1 | 16100 |
| **ACAT** | 12 | 1 | 84435 |
| **AAAAT** | 15 | 1 | 42399 |
| **AAATG** | 15 | 1 | 66748 |
| **ATATATC** | 21 | 1 | 36753 |
