# Supplementary material for: First chloroplast genomics study of Phoenix dactylifera (var. Naghal and Khanezi): A comparative analysis
Source: PLoS One. 2018 Jul 31;13(7):e0200104. doi: 10.1371/journal.pone.0200104 (PMC6067692; doi:10.1371/journal.pone.0200104)
Supplement: S9 Table — (DOCX) [file pone.0200104.s009.docx]

**S9 Table. Indel and SNP analysis of cp genomes from Khanezi an Naghal with other two date palm varieties**

| **Khanizi** | | | **Naghal** | | |
| --- | --- | --- | --- | --- | --- |
|  | **INDEL** | **SNP** |  | **INDEL** | **SNP** |
| **Naghal** | 35 | 23 | **Khanizi** | 35 | 23 |
| **Aseel** | 293 | 18 | **Aseel** | 292 | 10 |
| **Khalas** | 299 | 16 | **Khalas** | 296 | 12 |
