# Supplementary figures and images for: First chloroplast genomics study of Phoenix dactylifera (var. Naghal and Khanezi): A comparative analysis
Source: PLoS One. 2018 Jul 31;13(7):e0200104. doi: 10.1371/journal.pone.0200104 (PMC6067692; doi:10.1371/journal.pone.0200104)

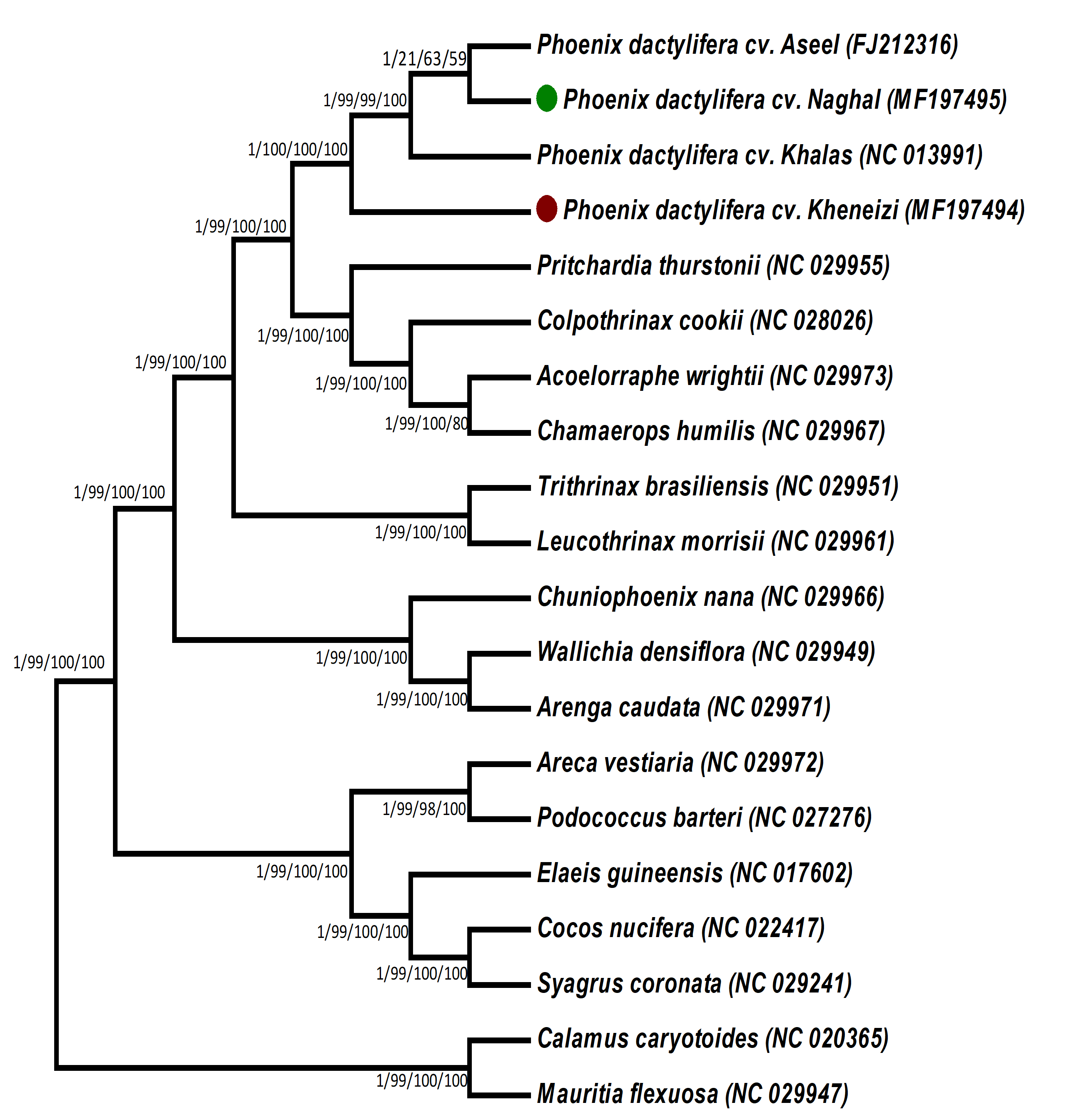

Supplement: S1 Fig — The following four methods were used for the entire genome data set: Bayesian inference (BI), maximum parsimony (MP), maximum likelihood (ML) and neighbor-joining (NJ). Numbers above the branches are the posterior probabilities of BI and bootstrap values for NJ, MP and ML. Green and brown dots represent the positions of P. dactylifera var Khanezi and Nagha. (TIF) [file pone.0200104.s011.tif]
